# Design and Experimental Validation of a 12 GHz High-Gain 4 × 4 Patch Antenna Array for $S_{21}$ Phase-Based Vital Signs Monitoring

**DOI:** 10.3390/s26030887

**Published:** 2026-01-29

**Authors:** David Vatamanu, Simona Miclaus, Ladislau Matekovits

**Affiliations:** 1 Doctoral School, Technical University of Cluj-Napoca, 400114 Cluj-Napoca, Romania; vatamanu.david@forter.ro; 2 Department of Communications, IT & Cyber Defense, “Nicolae Balcescu” Land Forces Academy, 550170 Sibiu, Romania; 3 Department of Electronics and Telecommunications, Politecnico di Torino, 10129 Turin, Italy; ladislau.matekovits@polito.it; 4 Istituto di Elettronica e di Ingegneria dell’Informazione e delle Telecomunicazioni, National Research Council of Italy, 10129 Turin, Italy; 5 Department of Measurements and Optical Electronics, Politehnica University Timișoara, 300006 Timișoara, Romania

**Keywords:** non-contact vital signs monitoring, microwave radar, *S*_21_ phase, patch antenna array, VNA-based radar, heartbeat detection

## Abstract

Non-contact monitoring of human vital signs using microwave radar has attracted increasing attention due to its capability to operate unobtrusively and through clothing or light obstacles. In vector network analyzer (VNA)-based radar systems, vital signs can be extracted from phase variations in the forward transmission coefficient $S_{21}$, whose sensitivity strongly depends on the electromagnetic performance of the antenna system. This work presents the design, optimization, fabrication, and experimental validation of a high-gain 12 GHz 4 × 4 microstrip patch antenna array specifically developed for phase-based vital signs monitoring. The antenna array was progressively optimized through coaxial feeding, slot-based impedance control, stepped transmission line matching, and mitered bends, achieving a simulated gain of 17.8 dBi, a measured gain of 17.06 dBi, a reflection coefficient of −26 dB at 12 GHz, and a total efficiency close to 74%. The antenna performance was experimentally validated in an anechoic chamber and subsequently integrated into a continuous-wave VNA-based radar system. Comparative measurements were conducted against a commercial biconical antenna, a single patch radiator, and an MIMO antenna under identical conditions. Results demonstrate that while respiration can be detected with moderate-gain antennas, reliable heartbeat detection requires high-gain, narrow-beam antennas to enhance phase sensitivity and suppress environmental clutter. The proposed array significantly improves pulse detectability in the (1–1.5) Hz band without relying on advanced signal processing. These findings highlight the critical role of antenna design in $S_{21}$-based biomedical radar systems and provide practical design guidelines for high-sensitivity non-contact vital signs monitoring.

## 1. Introduction

Non-contact detection of human vital signs using microwave and millimeter-wave radar has emerged as one of the most promising approaches for unobtrusive health monitoring in medical, rescue, and surveillance environments. Over recent decades, radar-based sensing of respiration and heartbeat has progressed from early continuous-wave (CW) Doppler concepts to highly integrated frequency-modulated continuous-wave (FMCW), ultra-wideband (UWB), and multi-antenna systems, offering enhanced resolution, robustness, and applicability across a broad range of distances and operating conditions [1,2,3,4,5,6]. Several studies have demonstrated the feasibility of extracting micro-motions of the chest cavity associated with respiration and heartbeat using Doppler, FMCW, leaky-wave, and UWB radars [7,8,9,10,11,12]. These techniques provide the ability to operate through clothing, bedding, and even certain wall materials [13], making them especially relevant in search-and-rescue and remote monitoring applications.

A specific category of vital-sign radars is the class of two-antenna systems driven by a vector network analyzer (VNA). In such systems, vital signs are extracted by tracking variations in the phase of the forward transmission coefficient $S_{21}$, which carries information about the relative motion between the transmitter, receiver, and the human thoracic surface [14,15]. VNA-based vital-sign detection presents several advantages:

(1) the phase of $S_{21}$ is typically more linear (far from resonance) and more stable compared to the reflection coefficient $S_{11}$, improving the extraction of micro-displacements;
(2) VNA instruments provide high dynamic range and frequency resolution;
(3) multi-frequency scanning enables characterization of the optimal operating bands for a given antenna configuration.

However, one of the major challenges of $S_{21}$-based detection is the strong dependence of system sensitivity on antenna performance, including gain, directivity, polarization, impedance matching, internal losses, and the mutual coupling between the transmitting (TX) and receiving (RX) antennas.

The antenna system performances therefore play a central role in the detectability of small thoracic motions, as it directly affects both the transmitted field strength and the stability of the received phase. Prior studies have shown that antenna parameters such as gain, radiation pattern, polarization, and reflection coefficient strongly influence radar sensitivity and noise robustness [3,7,16,17,18,19]. Likewise, antenna impedance characteristics, including surface currents, bandwidth, and the compensation of microstrip discontinuities, were shown to have direct implications for minimizing phase distortions [20]. Several works have also emphasized that optimal heartbeat detection often requires a careful balance between beamwidth and gain: narrow beams may improve signal-to-noise ratio (SNR) but increase sensitivity to subject displacement, while broader beams enhance robustness but reduce phase slope and effective sensitivity [21,22,23].

Over the years, numerous antenna types in single radiator configuration have been proposed for radar-based vital-sign detection: microstrip patch antennas [3,8], Sierpinski fractal designs [3], flexible liquid crystal polymer (LCP)-based antennas [24,25], horn antennas [2], substrate-integrated waveguide (SIW) cavity-backed [16,26], tapered slot and leaky-wave antennas [4,27], as well as high-frequency 77–120 GHz antennas capable of detecting subtle micro-motions [28,29,30]. Each antenna category introduces trade-offs between bandwidth, gain, mechanical rigidity, and fabrication complexity. For vital-sign detection based on Doppler effect and phase variations, these parameters must be globally optimized not only for efficient power transmission but also for stable phase response, low multipath interference, and minimal frequency-dependent group-delay ripple. Polarization purity further affects measurement quality, with circular polarization (CP) antennas offering robustness against subject rotation [21,22] and linear polarization offering potential advantages in controlled laboratory environments.

Recent studies have also focused on multi-antenna and array configurations to improve detection sensitivity and angular robustness. Arrays offer increased gain and beamforming capability [30,31], which can reduce clutter and improve isolation between TX and RX antennas—an essential aspect for accurate $S_{21}$ measurement. Advanced configurations, such as multi-layer leaky-wave antennas [4], tapered arrays [27], or wideband high-gain patches [32], have demonstrated improved performance in short-range biomedical radar systems. In addition, computational design tools have enabled the optimization of antenna topology and feed architecture for enhanced detection of micro-displacements (e.g., [33]).

Despite the substantial body of work on radar architectures and signal processing, comparatively few studies investigate in depth how antenna characteristics influence the sensitivity of phase-based measurements, especially when using VNA-driven $S_{21}$ acquisition. Most works concentrate on CW or FMCW radars with fixed RF front-ends. As a consequence, the precise relationship between antenna design—particularly impedance bandwidth, directivity, coupling, and near-field variations—and the detectability of micro-motions remains insufficiently quantified. Furthermore, the interaction between TX and RX antennas in VNA-based systems can introduce additional phase non-linearities that must be carefully minimized to enable accurate heartbeat extraction.

Addressing this gap, our previous work [34], provides a significant step toward understanding how a computational parametric study of the antenna system can increase detection sensitivity in Doppler radar configurations operating across UHF–SHF bands. The findings strongly support the hypothesis that antenna design plays a more critical role in vital-sign radar systems than generally acknowledged, especially when phase-based detection is employed. By integrating these insights with the capabilities of modern VNA-based systems, it becomes possible to design antenna pairs that maximize $S_{21}$ phase sensitivity while minimizing noise, reflections, and environmental interference.

Given this context, the present work focuses on achieving and optimizing the antenna system at 12 GHz for improved sensitivity in VNA-based $S_{21}$ vital-sign detection. Building upon the extensive research conducted in radar sensing, antenna engineering, and biomedical monitoring, this study aims to identify key antenna parameters—such as gain, directivity, beam overlap, polarization, impedance matching, and bandwidth—that directly influence the detectability of micro-movements associated with human respiration and heartbeat. By grounding the analysis in both theoretical understanding and evidence from prior work [1,4,11,22,24,34], this research contributes to a more systematic approach for designing antenna systems dedicated to high-sensitivity biomedical radar.

The selection of the 12 GHz operating frequency (X-band) is motivated by a strategic trade-off between detection sensitivity, penetration capability, and system compactness. Theoretically, the phase sensitivity to micro-displacements increases with frequency. Consequently, the 12 GHz band provides a wavelength (approx. 25 mm) short enough to generate significant phase variations even for micrometer-scale cardiac motions, which would be negligible at lower frequencies (e.g., 2.4 GHz). At the same time, this frequency allows for the design of a high-gain array, i.e., with high equivalent area, with a relatively small physical footprint, facilitating integration into portable devices—a constraint that is difficult to meet with high-gain antennas at lower frequencies. Although high-gain volumetric antennas, such as horn antennas, could theoretically be used for this application, they present significant drawbacks in terms of size, weight, and integration complexity. A horn antenna typically has a large longitudinal profile, making it unsuitable for compact, low-profile monitoring devices. In contrast, the microstrip patch array proposed in this work offers a planar space-effective solution with comparable gain.

To complete the research, the use of the designed antenna array for CW vital signs monitoring at 12 GHz is compared against other three types of antennas, among which, one is a commercial model. An extensive measurement campaign was carried out, and results were presented, with minimal emphasis on pre-or post-processing of the time series of raw data, with just classical Fourier extraction of the pulse and respiration being shown.

## 2. Design, Simulation, Optimization, and Prototyping of a High-Gain 4 × 4 Patch Antenna Array at 12 GHz

Our principal aim was to design and fabricate a planar antenna array, relatively simple and cheap, but with very high gain and directivity at 12 GHz, and with significant total efficiency.

The geometric arrangement of the elements influences the radiation pattern. The 4 × 4 planar arrays are used in many modern applications due to their ability to provide high gain, increased directivity, and beam control. Beam control in the antenna array is implemented through a beamforming network (BFN); however, when an equal-length BFN is employed, the resulting radiation pattern corresponds to broadside radiation. The geometric arrangement of the elements essentially influences the radiation pattern. Thus, linear arrays concentrate energy in a single plane, while a planar m × n array, such as 4 × 4, allows beam narrowing in both planes (azimuth and elevation), resulting in a more focused beam.

The 4 × 4 patch antenna arrays are extensively used in advanced communications applications, in the present case in the Ku band. Due to their capacity, they can offer high gain and increased directivity. Optimization involves action on the geometry of the patches and the feed structure, the dielectric substrate, and any auxiliary elements, such as cavities or parasitic metal elements—in case of more complex designs. A representative example is the antenna array made with Substrate-Integrated Waveguide (SIW) technology, where each patch in the array lays within a metallized cavity. This contributes significantly to increasing the quality factor and reducing mutual coupling between elements. In the case of 4 × 4 arrays optimized for the Ku band (9–13 GHz), a gain of up to 18.7 dBi was achieved, using Rogers Duroid 5880 substrates [16].

In another study, the application of parallel metallic plates along the array axis of a 4 × 1 array was investigated, resulting in a gain increase from 9.8 dBi to 16.8 dBi [17]. Similarly, in a 4 × 4 array, these plates can be adapted to enable improved beam steering and a narrower main-lobe beamwidth In addition, the use of semi-elliptical slot patches and cut-out mass structures allows for more efficient radiation control. In a 4 × 4 configuration operating at 28 GHz, the gain achieved was 16.54 dBi, and the radiation efficiency was over 80% [18]. In terms of configurations operating in thermally demanding environments (−50 °C to +150 °C), a 4 × 4 architecture based on double H-shaped slot patches achieved a gain of 18.7 dBi and a wide bandwidth centered at 15.5 GHz, demonstrating excellent stability [32].

In conclusion, a high performance 4 × 4 network configuration requires careful selection of the substrate (ideally with low losses), the use of additional structures (cavities, slots, reflector plates), and advanced design of the feed lines to maximize gain, efficiency, and bandwidth. These networks are essential for applications in 5G mobile communications, automotive radar, and satellite communications.

**Figure 1:**
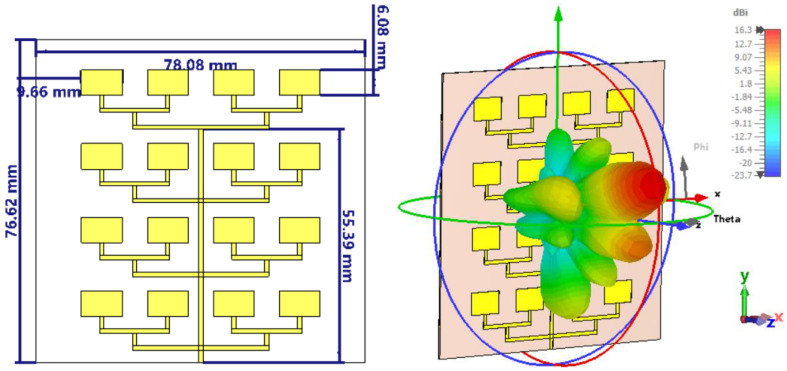
Initial geometric configuration of the 4 × 4 patch antenna array (**left**); simulated 3D radiation pattern at 12 GHz (**right**).

The patch antenna array under investigation here was designed for operation in the X/Ku band common limit, is centered at 12 GHz, and consists of 16 radiating elements arranged in 4 rows and 4 columns. The geometry of the initial configuration of the 4 × 4 antenna array is shown in Figure 1 (left), and the substrate used was RO-4350, chosen for its moderate relative permittivity and low losses. Each patch is connected via a corporate-type feed network, which distributes the signal to all 16 elements. The physical dimensions of the board are 78.08 mm × 76.62 mm, with a spacing between patches of approximately λ/2, designed to reduce mutual coupling and avoid the appearance of parasitic lobes.

The three-dimensional radiation pattern, obtained by simulation in CST Studio [33], is shown in Figure 1 (right). A clearly defined main lobe is observed, with a maximum gain of 16.3 dBi, but it is slightly tilted relative to the normal z-axis, indicating a possible phase imbalance in the network. In addition, several secondary lobes are visible in the lateral directions, indicating a phase imbalance inherent to the asymmetric planar feed network. Since the array is fed from the bottom edge, the signal path lengths to the upper and lower rows are not perfectly phase-compensated across the entire bandwidth, resulting in a slight beam squint. One should also note that, contrary to the usual location of the feeding point in the center of the geometry for an equal length configuration, the feeding here is from one side of the overall array. This choice will guarantee a higher polarization purity, since all patches are radiating with the same linear polarization without the need for a lambda/2 phase changer, which is necessary when half of the patches are feed in opposition of phase. However, the initial configuration requires further optimization to reduce secondary lobes and concentrate energy into a better-focused main beam.

Multiple techniques were used to optimize the antenna parameters:

A. ***Implementation of coaxial feeding in the antenna array***

**Figure 2:**
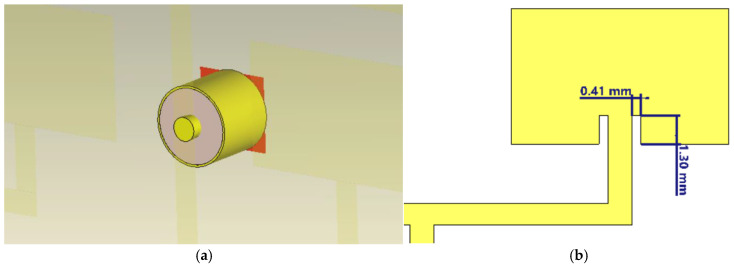
Detailed geometry of the antenna design elements: (**a**) 3D view of the coaxial-to-microstrip transition fed from the bottom ground plane; (**b**) top view of a single radiating element detailing the inset feed slot dimensions.

To improve impedance matching and achieve a more uniform phase distribution in the 4 × 4 network, the microstrip planar feed was replaced with a coaxial port feed mounted on the bottom of the substrate. This central feed method has a number of important advantages. First, it significantly reduces the complexity of the planar feed network by eliminating long, branched traces, which simplifies physical implementation and reduces conductor losses. It also helps improve feed symmetry by avoiding phase variations between patch elements and ensuring uniform signal distribution. With its central positioning and compact structure, this type of feed allows for efficient integration into dense networks, such as the 4 × 4 configuration. In addition, by eliminating extended microstrip portions, additional losses caused by the conductor and substrate are minimized. The main advantage of coaxial feed techniques is that the feed can be placed in any desired position within the patch to achieve impedance matching [35]. The specific geometric configuration of the coaxial-to-microstrip transition used in this design is illustrated in Figure 2a.

Compared to the previous version, there is a significant correction in the direction of the main lobe, which is now almost perfectly aligned with the axis normal to the antenna surface (Oz). In addition, the sides of the main beam are more balanced, and the secondary lobes are less pronounced. This behavior suggests uniform phasing of the signals in the network and more efficient impedance matching at the feed point.

The maximum gain obtained remains high, with a value of 16.5 dBi, and the radiation symmetry has improved compared to the planar feed case. This feed solution is thus more suitable for planar networks with central feeding, reducing parasitic influences and facilitating integration into compact radiating structures.

B. ***Insertion of slots in patch antennas***

To improve impedance matching and overall performance of the 4 × 4 array, a geometric adjustment was made to each patch in the form of symmetrical side slots, realizing a so-called inset fed. The geometric details of these slots, optimized to dimensions of 0.41 mm and 1.30 mm, are presented in Figure 2b. These slots act as impedance matching elements, allowing for more efficient matching between the feed line and the patch. In addition, they influence the distribution of currents on the surface of the radiators, providing additional control over bandwidth and radiation characteristics. An increase in antenna gain to 17.1 dBi was observed, along with improved directivity compared to previous versions. This result confirms the effectiveness of cutouts in optimizing network radiation.

**Figure 3:**
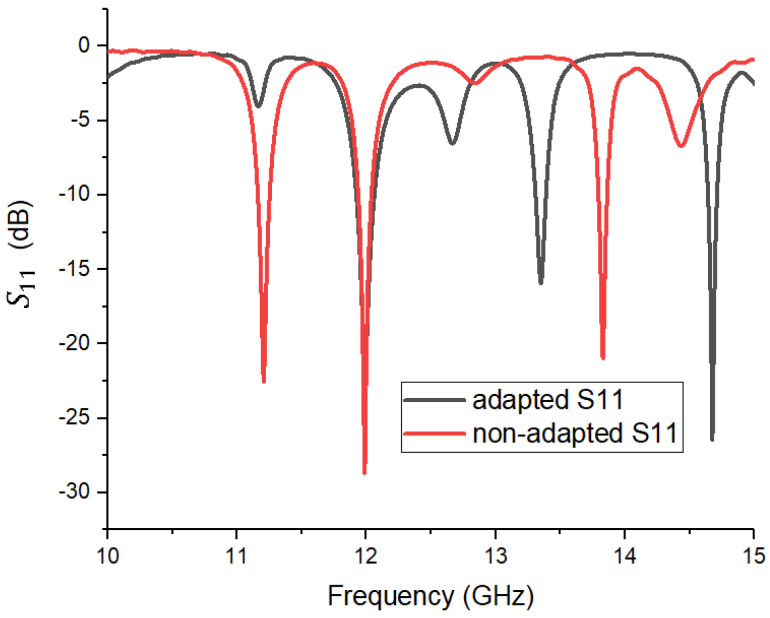
Reflection coefficient $S_{11}$ in the matched (black) and before optimization (red) cases.

Initially, optimization was performed exclusively based on considering the $S_{11}$ reflection coefficient, which led to an apparently good matching (Figure 3). However, further analysis of the antenna gain showed poor performance, with diffuse radiation and lack of directivity. This discrepancy highlighted that minimizing the reflection coefficient alone is insufficient if the input impedance does not converge to 50 Ω. Therefore, the conclusion of this step is that the introduction of the inset slots was necessary to align, as much as possible, the real part of the antenna’s input impedance with the 50 Ω feed line characteristic impedance. This true impedance matching maximized the power transfer efficiency, directly resulting in the observed gain increase.

C. ***Impedance matching by gradually changing the impedances on the sections of the feed line***

**Figure 4:**
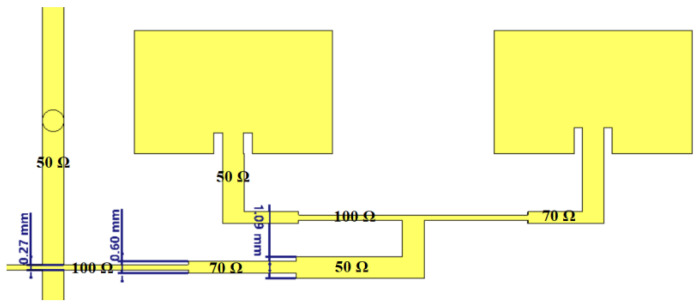
Step-up section of the feed line, with transitions between impedances of 50 Ω, 70 Ω, and 100 Ω.

To achieve an efficient matching between the source impedance and the input impedance of the 4 × 4 network, a stepped impedance matching strategy based on microstrip sections of different impedances was used. This method involves a progressive sequence of geometric transitions between 50 Ω, 70 Ω, and 100 Ω segments, designed to reduce reflections and ensure a gradual match along the entire length of the RF path. Figure 4 illustrates the structural details of the implemented matching section. This stepwise matching method is well documented in the literature.

Techniques based on controlled transitions between 100 Ω and 50 Ω impedances have been proposed to reduce the reflection coefficient of patch antennas, highlighting the critical role of progressive impedance matching [19]. In a different study, it was shown that gradually changing the width of the microstrip line allows for optimizing power transfer and minimizing losses over a wide range of impedances, which is essential in designing compact, high-performance structures [19].

**Figure 5:**
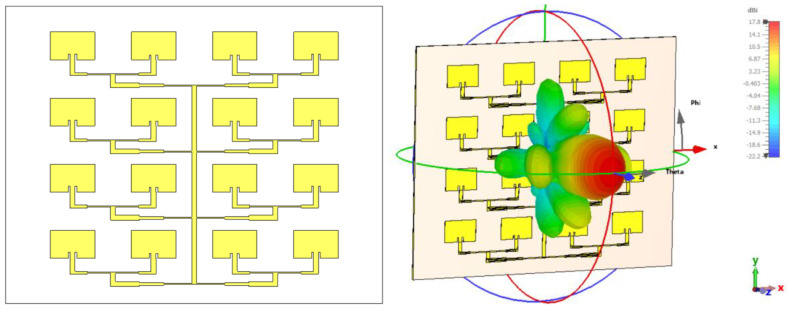
Antenna configuration with stepped impedance matching of the feed line (**left**); corresponding 3D radiation pattern (**right**).

In the present research, the reference impedance was 50 Ω, specific to most measuring and connecting equipment. For adaptation, two intermediate sections with impedances of 70 Ω and 100 Ω, respectively, were used, which reduced reflections and improved power transfer to the antenna elements. The widths of the microstrip lines were determined using standard semi-empirical formulas, in which the parameters specific to the RO-4350 substrate (relative permittivity and thickness of 0.5 mm) were introduced (Figure 5, left).

For *W*/*h* ≤ 1:

(1) $$Z_{0}=\frac{60}{\sqrt{\varepsilon_{eff}}}ln\left(8\frac{h}{W}+0.25\frac{W}{h}\right)$$

For *W*/*h* > 1:

(2) $$Z_{0}=\frac{120*\pi }{\sqrt{\varepsilon_{eff}}\frac{W}{h}\;+\;1.393\;+\;0.667ln\left(\frac{W}{h}\;+\;1.444\right)}$$

where $Z_{0}$ is the expected impedance, *W* is the line width, *h* is the substrate thickness, and $\varepsilon_{eff}$ is the effective permittivity.

With the implementation of this stepped adaptation, the maximum gain increased, confirming a more efficient impedance adaptation. The 3D radiation pattern (Figure 5, right) shows a better concentration of the main beam and an increase in directivity compared to previous variants without adaptation.

D. ***Additional optimization through oblique connection (tapping) along the bends of the supply line for fine impedance adjustment***

To improve impedance matching and more accurately control current distribution in the 4 × 4 network, additional optimization of the feedline geometry was introduced, consisting of oblique cuts or notches in the connection area between the microstrip lines and patches. These oblique connections, known as mitered bends, are used to compensate for the undesirable electrical effects generated by sharp corners (at 90 degrees), which can induce reflections, additional losses, or discontinuities in local impedance. The application of these mitered bends was inspired by the methodology proposed by Douville and James, in which the corner side is cut obliquely at an optimal angle, determined by the width of the trace and the thickness of the substrate [20]. In this context, the compensation parameter *X* is calculated according to the relationship:

(3) $$X=D(0.52+0.65e^{-1.35\frac{W}{h}})$$

where $D=W*\sqrt{2}\;$ represents the equivalent diagonal of a square corner, and *W* and *h* are the line width and substrate thickness. These adjustments have the effect of attenuating local capacitive effects and ensuring a better impedance match, contributing to the stabilization of the $S_{11}$ response and reduction in reflected waves in the network.

Although the maximum gain remained almost unchanged from the previous version, there was a 0.4% increase in total efficiency. The $S_{11}$ reflection coefficient was significantly improved, offering a −10 dB bandwidth of 198.5 MHz and reaching a minimum of −26 dB at 12 GHz, which indicates excellent impedance matching at the operating point.

**Figure 6:**
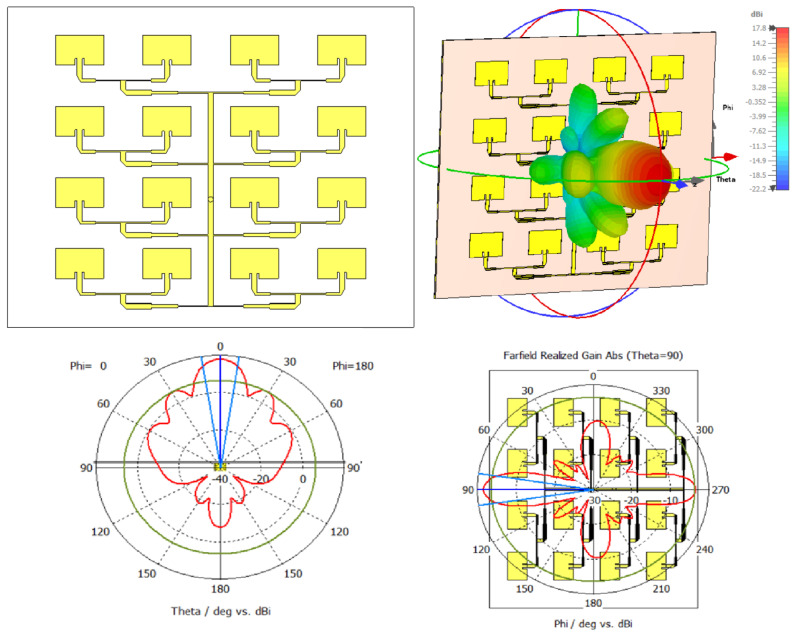
Final configuration of the optimized 4 × 4 array (**up-left**), the corresponding 3D radiation pattern (**up-right**), E-plane cut of the radiation pattern (**down-left**), and H-plane cut of the radiation pattern (**down-right**).

Figure 6 (up-left) shows the final configuration of the 4 × 4 patch array at 12 GHz, in which all the optimizations discussed above have been integrated. On the up-right side of Figure 6 is the 3D radiation pattern corresponding to this configuration. The far-field radiation characteristics are presented through 2D cuts of the 3D radiation pattern in the E-plane and H-plane, both passing through the main radiation direction, in the lower line of Figure 6.

**Figure 7:**
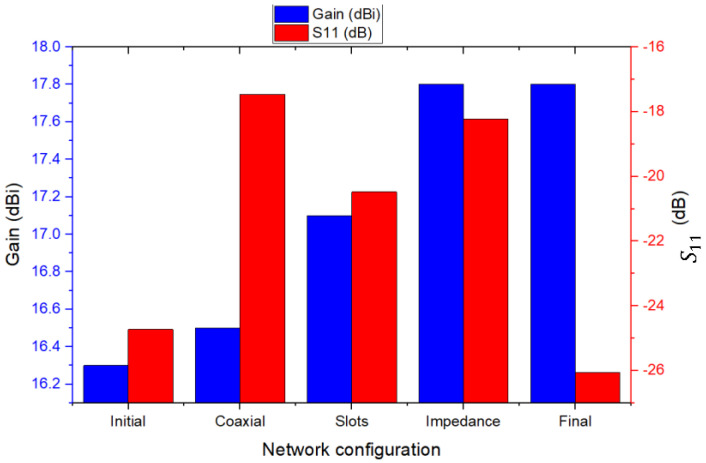
Evolution of gain (blue) and reflection coefficient $S_{11}$ (red) as a function of network configuration.

In order to summarize the results obtained from the progressive optimizations applied to the 4 × 4 array, a comparative Table 1 was created that presents the main design characteristics and measured or simulated performance for each configuration analyzed:

The evolution of performance can also be seen in Figure 7, which shows a comparison of the variation in gain and $S_{11}$ reflection coefficient for each optimization stage.

The configuration and progressive optimization of the 4 × 4 antenna array led to a significant increase in radiative performance. The antenna gain evolved from 16.3 dBi in the initial version to 17.8 dBi in the final configuration, reflecting a notable improvement in directivity and main beam concentration. At the same time, the reflection coefficient ($S_{11}$) reached a minimum value of −26.06 dB, indicating excellent impedance matching and very low reflected losses. At the same time, the efficiency of the system increased from 65.09% to nearly 74%, indicating an overall improvement in radiation quality. The implementation of slots and stepwise impedance matching proved essential for stabilizing operation in the 12 GHz band and maximizing power transfer, while the introduction of mitered bends helped reduce local reflections and achieve a more balanced electromagnetic feed.

**Figure 8:**
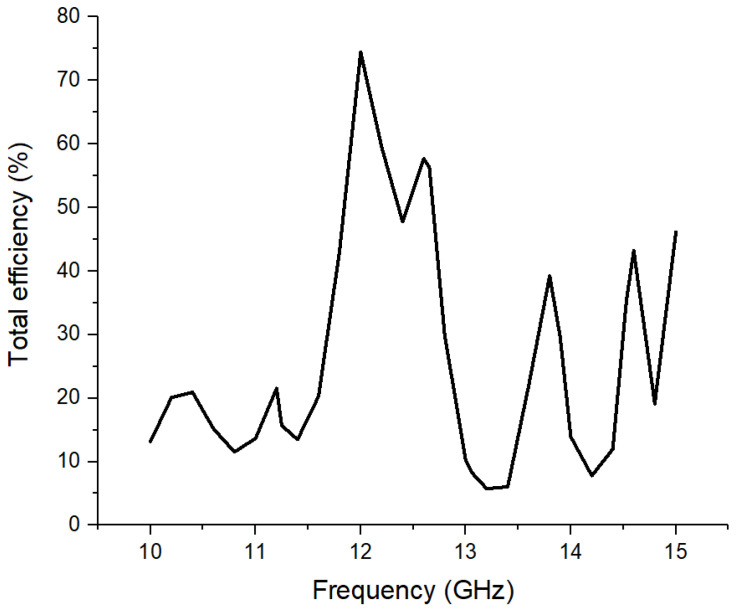
Variation in the simulated total efficiency of the 4 × 4 antenna array in the 10–15 GHz frequency band.

The simulated performance analysis is complemented by Figure 8, which illustrates the total simulated radiation efficiency of the optimized network in the 10–15 GHz frequency band. A pronounced maximum efficiency is observed, reaching a value of approximately 74% at the resonance frequency of 12 GHz. This result confirms that the ohmic losses in the conductors and the dielectric losses in the RO-4350 substrate, as well as the losses in the coaxial feed network, have been effectively minimized by the proposed design. High efficiency is critical in vital sign monitoring applications, where the detection of micro-movements of the chest depends directly on maximizing the power effectively radiated to the subject. The rapid decrease in efficiency outside the center frequency highlights the selective nature of the resonant structure, consistent with the previously observed impedance matching bandwidth.

**Figure 9:**
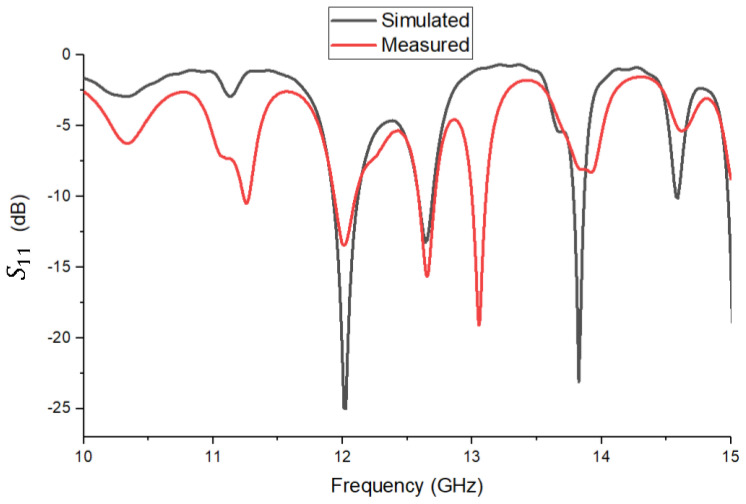
Comparison between simulated (black) and measured (red) $S_{11}$ reflection coefficient in the (10–15) GHz range.

To validate the impedance matching of the patch antenna prototype, the $S_{11}$ reflection coefficient was measured in the 10–15 GHz frequency band. The measurement was performed using a Keysight Technologies N5227A vector network analyzer under controlled conditions. Figure 9 shows the overlap between the curve obtained by simulation (black curve) and the experimentally measured result (red curve). There is a good overall correspondence between the two curves, with clear alignment in the main area of interest, around the frequency of 12 GHz. Both curves show a pronounced minimum at this point, which confirms the correct tuning of the antenna and the validation of the impedance matching in the designed band. In addition to the minimum at 12 GHz, the experimental measurement shows other minima at approximate frequencies of 11.25 GHz, 12.655 GHz, 13.055 GHz, 13.80 GHz, and 13.90 GHz. It can be seen that in the simulation, the minima are deeper and better defined, reaching values below −25 dB in the case of the center frequency. In contrast, in the measurement variant, the minimum values are less pronounced, reflecting the actual additional losses of the dielectric material, geometric tolerances, and environmental influences during testing. Overall, the curves obtained confirm that the theoretical model is well calibrated and that the design created in CST provides behavior close to the real one. Although there are small deviations in frequency and depth of the minima, these are within the limits accepted in the context of a practical application. It is remarkable that, although the simulation predicts a secondary resonance around 13 GHz, the measured gain at this frequency was only 9.29 dBi, much lower than the performance at 12 GHz. This confirms that the mode resonating at 13 GHz radiates inefficiently, i.e., having a minima in the broadside direction, corresponding to an asimetric feed for the two parts of the antenna. Therefore, it was not considered for operation, and its suppression in the measured $S_{11}$ curve is consistent with the actual losses and tolerances of the power supply network.

**Figure 10:**
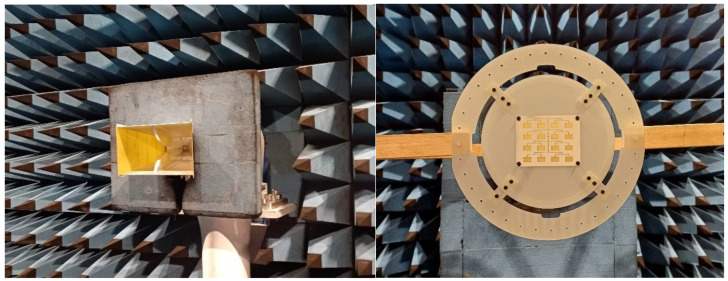
Rhode & Schwarz HF906 reference antenna (**left**). Prototype of the patch array antenna tested in the anechoic chamber (**right**).

To determine the actual radiation performance of the developed structure, the antenna was prototyped and its gain was measured in the far field, in a fully equipped anechoic chamber at the Polytechnic University of Turin, Italy. The method used was based on the comparison with a reference antenna, using a calibrated Rhode & Schwarz horn antenna model HF906, with known and well-defined characteristics in the analyzed frequency range. Figure 10 (left) shows the reference antenna used in the test, and Figure 10 (right) shows the physical prototype of the 4 × 4 patch antenna array fixed on the measurement support.

The antenna gain was calculated by processing the electric (*E*) field values recorded for two orthogonal polarization states: −90° and 0°, corresponding to the *E_φ_* and *E_θ_* components. For each polarization, the angle *θ* was scanned completely from 0° to 180°, while φ was varied in 2° steps from 0° to 178°. This measurement flow allowed the spatial distribution of radiation to be reconstructed by vector composition of the two orthogonal components of the *E*-field strength. For each frequency in the set 11.250, 12.000, 12.655, 13.055, 13.800, and 13.900 GHz, the amplitudes and phases corresponding to the two components were extracted.

To determine the gain of the designed antenna, a comparative method was used, based on the ratio of the *E*-field strengths radiated by two different antennas—one unknown and one reference—measured under the same conditions. The working hypothesis assumes that both antennas are powered with the same power, and the measurement is made in the same direction and at the same distance *r*, under far-field conditions. In order to obtain the value of the total field strength, *E*, radiated in the main direction, the orthogonal components *E_θ_* and *E_φ_* corresponding to the two polarization components were combined. The total magnitude of the electric field was determined according to the relation:

(4) $$E=\sqrt{{E_{\theta }}^{2}+{E_{\varphi }}^{2}}$$

This formula reflects the sum of the two perpendicular components, according to the theory of radiation in the far field regime, and is similar to the way electromagnetic solvers (such as HFSS or CST) work. By applying this relationship, a physically correct estimate of the amplitude of the radiated electric field was obtained, independent of the phase differences between the orthogonal components. According to the fundamental expression of the *E*-field radiated by an antenna in the maximum direction:

(5) $$E=\frac{\sqrt{30GP_{t}}}{r}$$

where *E* is the amplitude of the radiated electric field (in V/m), *G* is the antenna gain, $P_{t}$ is the supply power (*W*), and r is the distance to the measurement point (m).

If the values of $P_{t}$ and *r* are kept constant for both antennas, the ratio of the measured fields becomes directly proportional to the square root of the ratio of the gains:

(6) $${\left(\frac{E_{ant}}{E_{ref}}\right)}^{2}=\frac{G_{ant}}{G_{ref}}$$

where $E_{ref}$ is the amplitude of the *E*-field received by the reference antenna, $E_{ant}$ is the amplitude of the received *E*-field, $G_{ref}$ is the known gain of the reference antenna (in this case, the Rohde & Schwarz HF906 horn antenna), and $G_{ant}$ is the unknown gain of the tested antenna.

This results in the relationship meant for determining the gain of the tested antenna:

(7) $$G_{ant}=G_{ref}{\left(\frac{E_{ant}}{E_{ref}}\right)}^{2}$$

**Figure 11:**
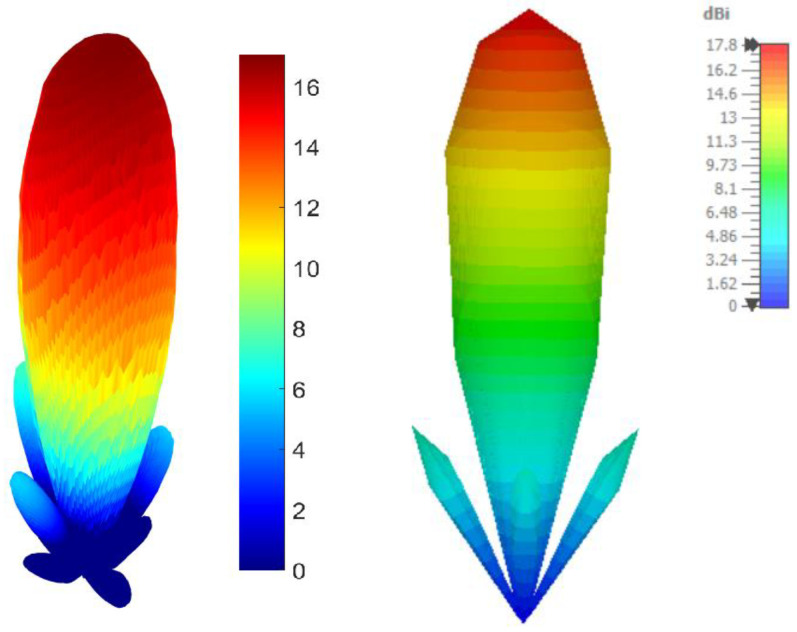
Three-dimensional radiation pattern comparison: measured one (**left**) vs. simulated one (**right**), at 12 GHz.

Figure 11 provides a visual comparison between the experimentally obtained 3D radiation pattern of the antenna gain (left) and the one generated by electromagnetic simulation (right). In terms of spatial distribution, there is a very good overlap between the two results, both showing dominant radiation in the vertical direction, with well-defined secondary lobes and corresponding symmetry of the feed system. The maximum measured gain is 17.06 dBi, compared to 17.83 dBi in the simulation, with a difference in less than 0.8 dB indicating a very good correlation and a faithful practical implementation of the theoretical model.

**Figure 12:**
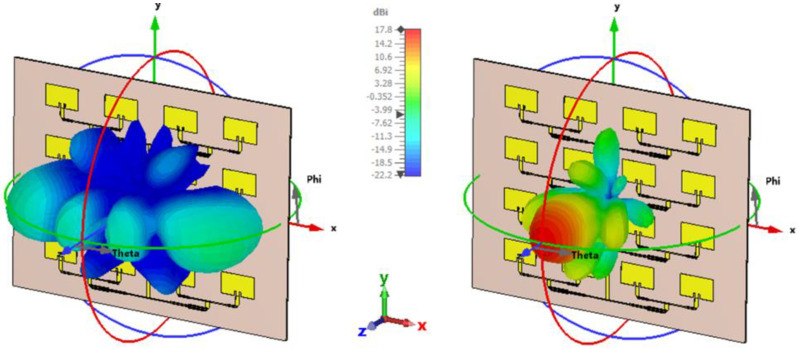
Simulated 3D radiation pattern for polarizations: (**left**) Cross-polarization; (**right**) Co-polarization component.

The co- and cross-polarization radiation patterns are used to evaluate the polarization purity of the antenna, as well as to assess undesired radiation components that may degrade system performance. Therefore, we represented these characteristics by using the Ludwig 3 coordinate system in CST Studio Suite. Figure 12 illustrates the spatial distribution of these components at 12 GHz. The image on the right (co-polarization) confirms a high gain and a well-defined main lobe oriented on the z-axis, with a peak value of 17.8 dBi. In contrast, the cross-polarization component (left image) shows significantly lower levels, with secondary lobes distributed predominantly in the diagonal planes, a behavior typical to patch antennas. Comparative analysis of the two diagrams indicates excellent polarization discrimination in the direction of maximum radiation (boresight). The large difference in levels between the co-polarized and cross-polarized components (over 20 dB in the simulation) demonstrates the network’s ability to maintain stable linear polarization. Additionally, the minimum value of the cross-pol term for theta = 0 deg indicates a good alignment of the antennas during the measurements.

## 3. Analyzing the Antenna Parameters Impact on Breath and Pulse Detection Sensitivity

Many current studies rely heavily on complex algorithms to clean noisy data; in contrast, we will demonstrate here, experimentally, that the true sensitivity limit is defined by the antenna’s physical characteristics and the VNA’s configuration. Our specific contributions will move beyond simply validating a single design. By systematically testing four different antenna types under identical conditions, we will provide empirical proof that heartbeat detection in S21-mode is physically impossible without high-gain, narrow-beam antennas, regardless of the signal processing used. Consequently, we will validate a ‘hardware-first’ approach through a planar 4 × 4 array that offers the performance of a bulky horn antenna but in a form factor suitable for integration, challenging the standard use of low-gain antennas in compact devices. Furthermore, we will identify that standard VNA settings are often suboptimal for biological signals, and we will establish a specific configuration protocol (optimizing IFBW vs. sampling rate) that is critical for capturing the transient nature of the pulse, a detail often overlooked in similar literature.

When the phase of the $S_{21}$ transmission coefficient at 12 GHz is employed for non-contact physiological monitoring, both respiration and cardiac activity modulate the received electromagnetic signal through subtle mechanical displacements and variations in the effective dielectric properties of biological tissues. Although both phenomena are observable in the phase domain, their physical origin, amplitude, and interaction mechanisms with microwave radiation differ substantially, resulting in markedly different detection sensitivities.

Respiration is primarily associated with the periodic expansion and contraction of the thoracic cavity and lungs, producing relatively large-amplitude, low-frequency chest wall displacements. Typical respiratory frequencies range from 0.1 to 0.4 Hz (6–24 breaths per minute). In contrast, cardiac activity originates from cyclic blood volume variations and arterial wall motion, generating very small, rapid mechanical vibrations, often localized in superficial vessels. The corresponding frequency content typically lies between 0.8 and 2 Hz (48–120 beats per minute). As a result, pulse detection represents a significantly more challenging task, requiring higher signal-to-noise ratio (SNR), increased phase stability, and finer temporal resolution compared to respiration monitoring.

At an operating frequency of 12 GHz, additional challenges arise from the presence of clothing layers between the antenna system and the subject. Textile materials introduce frequency-dependent attenuation and phase delay, governed by fabric thickness, dielectric constant, and loss tangent. While respiratory motion, due to its relatively large displacement, remains detectable even in the presence of clothing, pulse-induced arterial motion produces micrometer-scale phase perturbations that can be severely attenuated or masked. Consequently, reliable pulse detection through clothing at 12 GHz necessitates high-sensitivity phase measurements, antennas with sufficient gain and spatial selectivity, and careful mitigation of environmental and instrumental noise sources.

In practical implementations, the extraction of pulse information from the $S_{21}$ phase signal further requires dedicated signal processing steps. These typically include phase unwrapping to resolve discontinuities exceeding 2π band-pass filtering to separate respiratory (0.1–0.5) Hz and cardiac (0.8–2.0) Hz components from static clutter, and spectral analysis methods such as the fast Fourier transform (FFT) or time–frequency techniques. However, even with advanced processing, the quality of the extracted vital signs remains fundamentally constrained by the electromagnetic performance of the antenna system.

**Figure 13:**
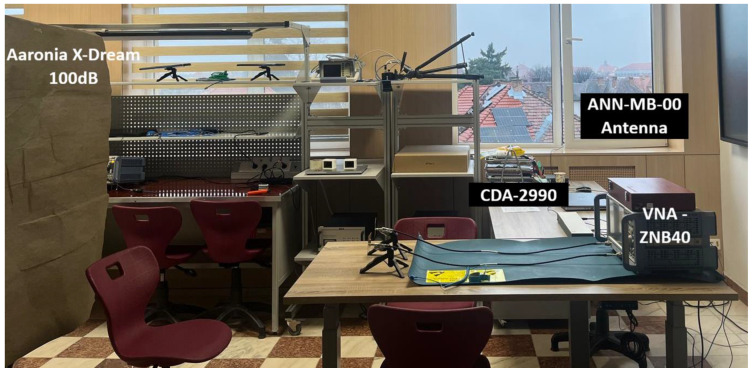
Experimental setup used for the comparative analysis of antenna sensitivity in vital signs detection.

To experimentally validate these considerations and to quantify the influence of antenna characteristics on detection sensitivity, a series of comparative measurements was conducted using the experimental setup illustrated in Figure 13. All subsequent tests were performed under identical conditions to ensure a fair comparison between different antenna configurations.

The measurement system was based on a Rohde & Schwarz ZNB40 VNA (Beverly Hills, CA, USA) operating in continuous-wave (CW) mode at 12 GHz. Given the stringent phase stability requirements imposed by micrometer-scale motion detection, the internal time reference of the VNA was externally synchronized using a CDA-2990 clock distribution amplifier connected to a precision GNSS antenna (ANN-MB-00, u-blox, Thalwil, Switzerland) placed outside the room, near the window. This synchronization architecture significantly reduced phase noise and long-term oscillator drift. The antenna pair was positioned at a distance of 1 m from the subject (far field condition), while an RF absorbing shield (Aaronia X-Dream, Aaronia, Strickscheid, Germany, 100 dB attenuation) was placed behind the subject to suppress multipath reflections and wall-induced interference. This semi-controlled environment enabled a systematic comparison of the proposed 4 × 4 patch array with alternative antenna topologies, including a commercial broadband biconical antenna, a single patch radiator, and an MIMO antenna structure. The high directivity of the 4 × 4 array plays a crucial role in reducing/suppressing lateral radiation, thereby enhancing isolation. Furthermore, the VNA was calibrated prior to measurements to remove systematic errors, including the static direct coupling between the ports.

### 3.1. Comparison Between 4 × 4 Patch Array and Commercial Biconical Antenna at 12 GHz

**Figure 14:**
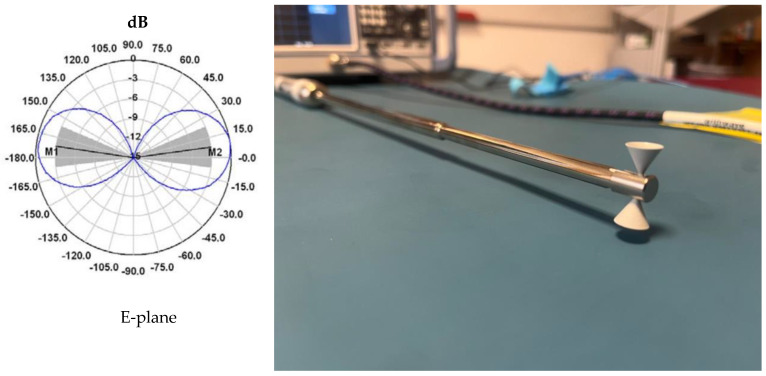
(**left**) Characteristic radiation pattern in the E plane at a frequency of 12 GHz; (**right**) Schwarz beck SBA 9112 broadband biconical antenna.

To evaluate the effectiveness of the designed patch antenna array in the context of vital sign monitoring, a comparative analysis was performed using a commercial antenna as a reference. The reference antenna chosen is the Schwarzbeck SBA 9112 biconical antenna, known for its wide bandwidth and stable frequency characteristics. This antenna has a modest isotropic gain of 1.17 dBi and a typical voltage standing wave ratio (VSWR) of 1.6 at the operating frequency of 12 GHz. Its omnidirectional radiation pattern in the H-plane (shown in Figure 14) makes it ideal for comparison with the directional array antenna.

**Figure 15:**
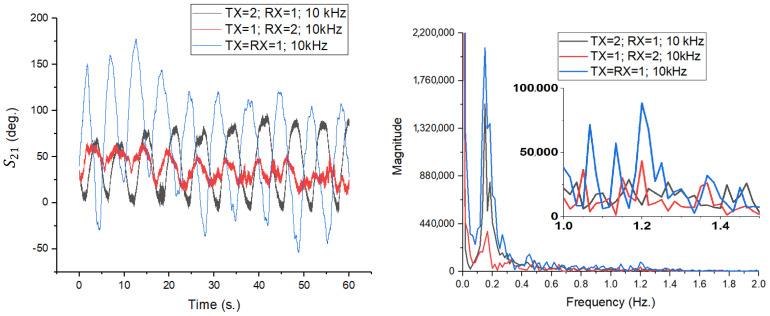
Time-domain variation in the $S_{21}$ phase (**left**) and FFT amplitude spectrum (**right**) for the three configurations analyzed at IFBW = 10 kHz.

The impact of antenna directivity on detection sensitivity was quantified and reported in Figure 15, through three distinct measurement scenarios, maintaining the same intermediate frequency filter bandwidth (IFBW) of 10 kHz over a duration of 60 s:

- TX = RX = 1 (Blue Line): Transmission and reception using the 4 × 4 patch antenna array.
- TX = 2; RX = 1 (Black Line): Transmission with the biconical antenna, reception with the patch array.
- TX = 1; RX = 2 (Red Line): Transmission with the patch array, reception with the biconical antenna.

The results in the time domain, illustrated in Figure 15 (left), demonstrate a major difference in the detected phase variation. The TX = RX = 1 configuration (both antennas being patch array type) shows the highest phase excursion, with the signal having a peak-to-peak amplitude of approximately 150–180 degrees. This high sensitivity is due to the high gain of the array (17.6 dBi). In contrast, mixed scenarios involving the biconical antenna (black and red lines) show a significantly attenuated signal amplitude and a higher noise level. Since the biconical antenna radiates omnidirectionally, much of the transmitted energy does not reach the subject, and at reception, it captures parasitic reflections from the surrounding environment, degrading the signal-to-noise ratio (SNR).

The spectral analysis of the signals, obtained by applying FFT (with no other pre-processing), is shown in Figure 15 (right). It can be seen that respiratory movement (low frequency component, 0.2–0.4 Hz) is detectable in all three cases, although the magnitude is significantly higher when using patch antennas exclusively.

Nevertheless, the critical difference appears in the detailed analysis of the spectral components associated with cardiac activity (1.0–1.5 Hz range), presented in the inset panel. In the TX = RX = 1 configuration (blue line), the spectral peaks corresponding to the pulse are clearly defined and distinctly distinguishable from the noise level. In contrast, for configurations using the biconical antenna (red and black lines), the cardiac components are strongly attenuated, often masked by the system noise threshold. This observation validates the hypothesis that the use of high-gain, narrow-beam antennas is essential for the reliable extraction of low-amplitude vital signs (pulse), while respiration, which has a much greater chest displacement, can also be monitored with less directional systems.

### 3.2. Comparison of 4 × 4 Patch Array—Patch Antenna at 12 GHz

**Figure 16:**
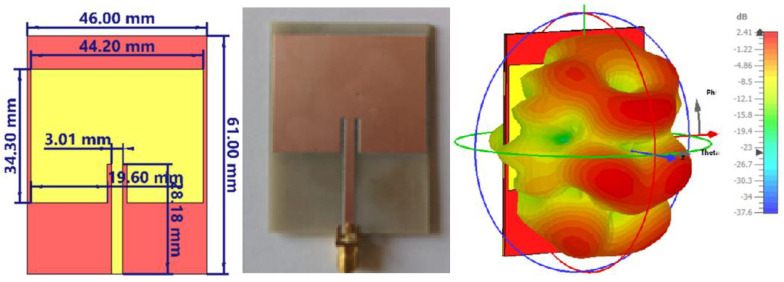
(**left**) Geometric dimensions of the simple patch antenna used for comparison; (**center**) Prototype of the simple patch antenna with SMA connector used in measurements; (**right**) Radiation pattern.

In order to separate and quantify the specific contribution of the array architecture to detection sensitivity, a direct comparison was made between the proposed array and its basic component: a single patch antenna. The reference antenna, shown in Figure 16, is a rectangular inset-fed radiator. This simple antenna provides a typical gain of approximately 2.41 dBi and a much wider beamwidth compared to the 4 × 4 array, with horizontal multi-lobes radiation pattern.

The impact of switching from a single radiating element to a phased array on the $S_{21}$ phase signal quality was analyzed through three test scenarios, maintaining the previous experimental conditions (IFBW = 10 kHz, body distance of 1 m):

- TX = RX = 1 (Blue Line): Complete system using 4 × 4 arrays for transmission and reception.
- TX = 1; RX = 3 (Red Line): Transmission with 4 × 4 array, reception with single patch antenna.
- TX = 3; RX = 1 (Black Line): Transmission with single patch antenna, reception with 4 × 4 array.

**Figure 17:**
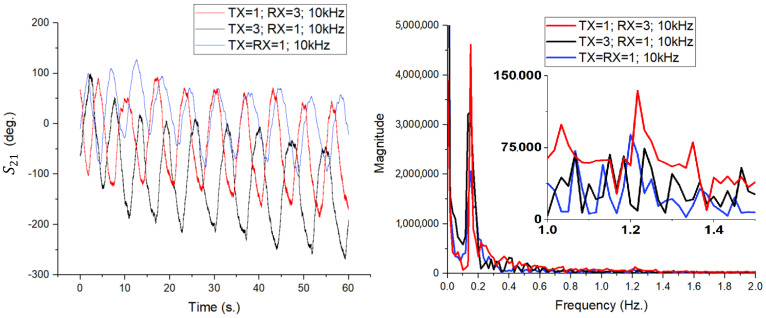
Time-domain variation in the $S_{21}$ phase (**left**) and FFT amplitude spectrum (**right**) for the three configurations analyzed at IFBW = 10 kHz.

The time domain results, shown in Figure 17 (left), demonstrate the system’s capability to extract respiratory motion with high clarity in all three configurations. Analysis of the waveforms indicates that there is no degradation in signal quality in the mixed configurations (red and black lines) compared to the reference configuration (blue line). The sinusoid associated with respiration is well defined and clean (without pronounced phase noise) in all cases.

Analyzing the detail window focused on the cardiac activity area (1.0–1.5 Hz), the TX = 1; RX = 3 configuration (red line) remains the most sensitive, with the pulse being extremely evident and clearly delimited from noise. Cardiac activity is also detectable in the standard configuration TX = RX = 1 (blue line), although with a slightly lower amplitude. In contrast, for the TX = 3; RX = 1 scenario (black line), the spectral components of the pulse are not visible, being completely masked by the system’s noise threshold, which indicates the limitations of using a simple patch antenna for detecting fine micro-movement due to the heart.

A more pronounced distinction is observed in the cardiac frequency band (approximately (1.0–1.5) Hz), in the TX = RX = 1 configuration, where the pulse-related spectral components are clearly resolved above the noise floor, enabling reliable heart rate extraction. When antenna 1 is used either at transmission or reception, these components are significantly attenuated and, in some cases, approach the system noise threshold. This behavior highlights the critical role of antenna gain and effective aperture in detecting micrometer-scale arterial motion, which induces phase variations close to the sensitivity limits of the measurement system.

Importantly, the asymmetry between the TX = 3; RX = 1 and TX = 1; RX = 3 configurations emphasizes the distinct roles of the transmitting and receiving antennas in phase-based sensing. While higher transmit gain increases the amplitude of the motion-induced phase modulation, higher receive directivity is particularly effective in suppressing environmental clutter and stabilizing the measured phase. This observation suggests that receive-side directivity plays a dominant role in enabling reliable pulse detection, although optimal performance is achieved when high-gain antennas are employed at both ports.

In summary, the comparison between the 4 × 4 patch array (antenna 1) and the single patch antenna (antenna 3) demonstrates that moderate-gain antennas are sufficient for respiration monitoring but inadequate for robust heartbeat detection at 12 GHz. The significantly higher gain and narrower beamwidth of the patch array provide a decisive improvement in phase sensitivity and clutter suppression, enabling reliable extraction of cardiac micro-motions. These findings further confirm that antenna design is a key enabling factor in non-contact vital sign monitoring systems based on $S_{21}$ phase measurements.

### 3.3. Comparison of 4 × 4 Patch Array—MIMO Antenna at 12 GHz

**Figure 18:**
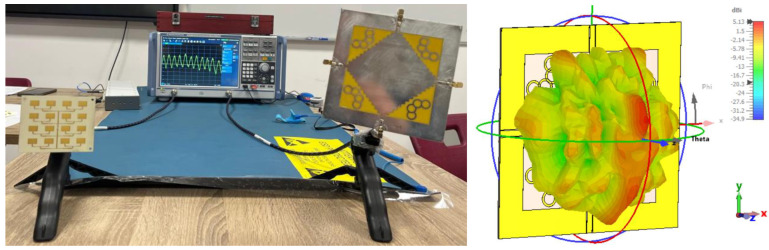
Experimental arrangement for comparing radiating performances: (**left**) 4 × 4 patch antenna on one port of VNA and MIMO antenna used as reference, at the other port; (**right**) Radiation pattern of the MIMO antenna at 12 GHz [36].

The final stage of the comparative analysis involved evaluating the 4 × 4 patch antenna array in relation to an antenna designed for MIMO systems. The antenna used as a reference, visible in Figure 18 with 4 ports (of which only 1 port was connected to the VNA), is a complex planar radiating structure (fractal/wideband type) [36], characterized by a simulated gain of 5.1 dBi and an $S_{11}$ reflection coefficient of −8.2 dB at a frequency of 12 GHz. Although its gain is lower than that of the 4 × 4 array, it is superior to the simple patch element analyzed previously, offering a different compromise between directivity and spatial coverage. Its radiation pattern (Figure 17, right) shows a highly lobulated radiation distribution, with large incident spatial coverage. The experimental setup, shown in Figure 18, maintains the 1 m test distance, with the MIMO antenna connected alternately to the VNA ports to generate the three test scenarios (TX = 4 denotes the MIMO antenna in the graph legends, for consistency with previous notations):

- TX = RX = 1 (Blue Line): Reference Array-Array configuration.
- TX = 4; RX = 1 (Black Line): Transmission with MIMO antenna, reception with patch array.
- TX = 1; RX = 4 (Red Line): Transmission with patch array, reception with MIMO antenna.

**Figure 19:**
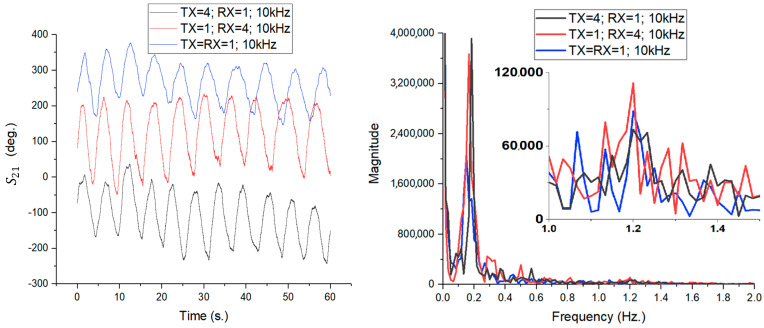
Time-domain variation in the $S_{21}$ phase (**left**) and FFT amplitude spectrum (**right**) for the three configurations analyzed at IFBW = 10 kHz.

The time domain analysis of the $S_{21}$ phase signal, shown in Figure 19 (left), reveals an interesting dynamic. All three configurations produce a clear sinusoidal waveform corresponding to breathing, without major distortions. The spectral examination in Figure 19 (right) provides a detailed overview of the sensitivity. In terms of breathing (low-frequency component, ~0.2 Hz), the mixed configuration TX = 4; RX = 1 (black line) shows the highest spectral magnitude, indicating superior sensitivity to large chest movements. This is closely followed by the reverse configuration (red line). Surprisingly, the symmetric array–array configuration (blue line) displays the lowest amplitude for respiration in this scenario, although the signal remains perfectly visible. This behavior can be explained by the narrow beam of the patch array, which, in the TX = RX configuration, scans a smaller area of the chest, while the wider multi-beam of the MIMO antenna (in mixed configurations) integrates movement over a larger body surface area. The situation changes in the case of pulse detection (1.0–1.5 Hz range, detailed in the inset). Here, all three configurations successfully extract the heart rate, with spectral peaks clearly distinguishable from noise. Thus, the TX = 1; RX = 4 configuration (red line) provides the clearest pulse detection, followed by the symmetrical TX = RX = 1 configuration (blue line). The configuration with the MIMO antenna at transmission (black line) ranks third, but still maintains a valid detection. This set of experiments demonstrates the robustness of the designed network. Even in hybrid scenarios, combined with low/medium gain antennas (such as MIMO), the system maintains the ability to detect fine vital signs (pulse), a performance that was not achieved when using the simple patch element or biconical antenna.

## 4. Experimental Analysis of the Detection Sensitivity of the 4 × 4 Patch Array Antenna

**Figure 20:**
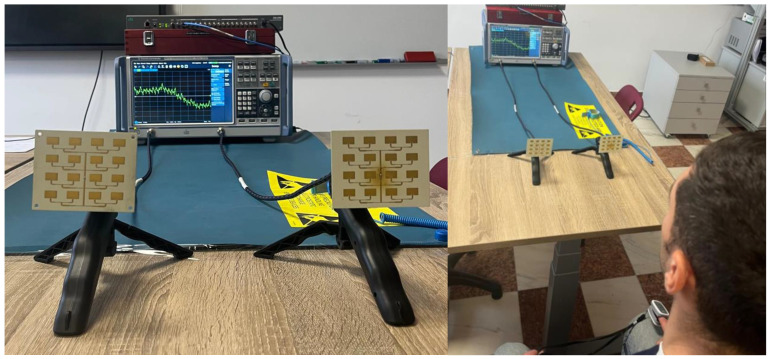
(**left**) Radar system consisting of two identical 4 × 4 patch arrays oriented towards the target; (**right**) Positioning of the human subject in the antenna radiation zone, monitored simultaneously with a reference sensor (pulse oximeter) for data validation.

After validating the performance of the 4 × 4 patch antenna array compared to the other radiating topologies, this section focuses on maximizing the sensitivity of the system by tuning the acquisition parameters of the VNA. The experimental setup used for this step is illustrated in Figure 20 and is based on the symmetrical TX = RX = Array architecture, which has been shown to provide the best phase stability. Non-invasive detection of vital signs by monitoring phase variations in the $S_{21}$ parameter involves a fine compromise between sampling rate (necessary to capture fast details of the cardiac waveform) and background noise reduction. The overall performance of the CW radar system does not depend entirely on the antenna gain, but is critically influenced by the internal settings of the VNA. In this context, the experimental approach aims to quantify the influence of three fundamental parameters on the quality of the extracted physiological signal:

- Number of acquisition points (temporal resolution): Determining the optimal sampling density to avoid undersampling of peaks without generating excessive amounts of data.
- IFBW: Analysis of the impact of this parameter on the phase noise level and sweep time.
- Acquisition duration: Evaluation of the long-term stability of measurements and identification of the optimal time window for robust monitoring.

The ultimate goal of this analysis is to define a set of configuration parameters that allow reliable pulse and respiration detection, minimizing errors introduced by the sampling and filtering process of the measuring instrument.

### 4.1. Analysis of the Impact of the Temporal Resolution of the Acquisition on the Amplitude of the Detected Pulse

The number of acquisition points per sweep is one of the critical parameters in configuring a VNA-based radar system. It directly determines the effective sampling rate and, implicitly, the system’s ability to accurately reconstruct fast waveforms. Although respiratory movement is slow and ample, heartbeats involve rapid dynamics, which risk being attenuated or lost in the event of undersampling.

To quantify this effect, two distinct scenarios were analyzed, setting the IFBW value at 100 kHz, 500 kHz, and 1 MHz:

- Case 1: Low resolution, set to 20,000 points per acquisition.
- Case 2: High resolution, set to 100,000 points per acquisition.

**Figure 21:**
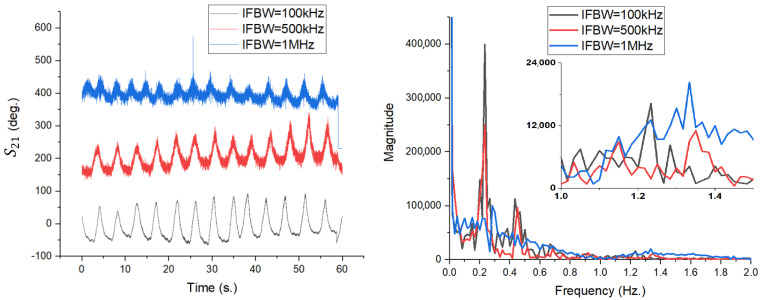
Signal analysis for the acquisition with 20,000 points: (**left**) Phase variation over time; (**right**) FFT spectrum, where the pulse components show reduced magnitude due to undersampling.

Figure 21 illustrates the results corresponding to the first scenario (20,000 points). In the time domain (left), the respiratory signal is visible for all three IFBW values, but the visible overlapping noise (which actually contains the cardiac information) is poorly defined. Spectral analysis (right) shows that although there are spectral components in the pulse region, their magnitude is relatively small. For example, for IFBW = 1 MHz (blue curve), the spectral amplitude of the pulse reaches a modest value, suggesting that at this resolution, the measurement points are not dense enough to capture the maximum phase excursion caused by the heartbeat.

**Figure 22:**
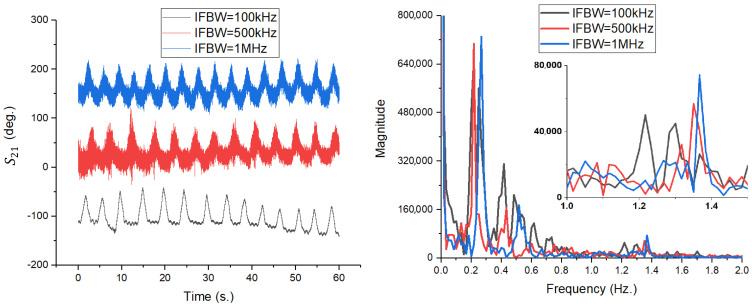
Signal analysis for the 100,000-point acquisition. (**left**) The waveform in the time domain is much more detailed; (**right**) The FFT spectrum shows a massive increase in pulse amplitude compared to the previous case.

The significant change in performance is highlighted in Figure 22, where the number of points has been increased to 100,000. In the time domain (left), the high density of samples allows for a much more accurate reconstruction of fine variations. This translates directly into the frequency spectrum (right), where the peaks corresponding to the pulse become prominent. Comparing the two cases directly, it can be seen that simply increasing the number of points (while keeping the total measurement duration constant) leads to a significant increase in the magnitude of the detected heart signal. This phenomenon is detailed quantitatively in Table 2.

The data in Table 2 reveal a direct correlation between sampling density and detection sensitivity. Detection sensitivity is quantified as the minimum peak-to-peak phase deviation in $S_{21}$ for which the corresponding heart rate and respiration frequency can be detected with a predefined SNR. For example, at an IFBW of 1 MHz, the transition from 20,000 to 100,000 points generated an increase in pulse magnitude from 20 × 10^3^ to 76 × 10^3^—an amplification of nearly 3.8 times. The physical explanation lies in the transient nature of the cardiac signal. The displacement of the chest wall caused by the heartbeat is a rapid impulsive event. If the time interval between two consecutive measurement points is too long (Case 1), the probability that the VNA will measure exactly at the moment of maximum extension is low, resulting in an averaging that flattens the signal peak. By increasing the number of points (Case 2), the temporal resolution becomes fine enough to capture the actual peak of the micro-movement, thus maximizing the signal-to-noise ratio and detection accuracy. Therefore, it is concluded that for monitoring rapid vital signs (pulse), the use of a high number of acquisition points (e.g., 100,000) is mandatory, even if this involves processing larger data sets.

### 4.2. The Effect of IFBW Change on Phase Tracking in Respiration and Pulse Detection

**Figure 23:**
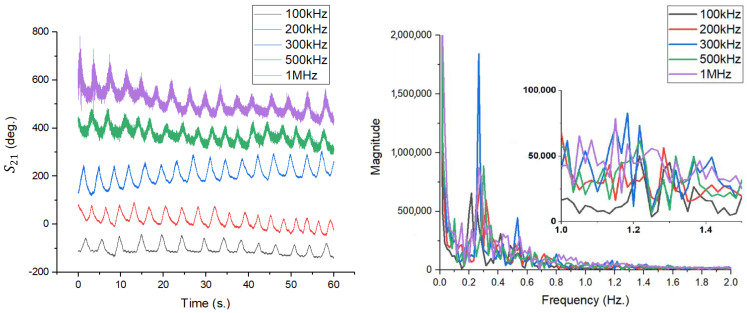
Analysis of the impact of IFBW on the $S_{21}$ signal. (**left**) Time domain signals show an increase in noise with increasing IFBW (purple curve vs. black curve); (**right**) FFT spectrums confirm that the 300 kHz setting (blue curve) maximizes the signal-to-noise ratio.

In the architecture of a VNA, the bandwidth of the intermediate frequency filter, IFBW, plays a dual role: it determines the noise floor and the acquisition speed. A narrow bandwidth reduces noise but increases the measurement time per point, risking smoothing out (by averaging) rapid signal variations. On the other hand, a wide bandwidth allows for fast acquisition but introduces a high level of broadband noise that can mask micro-vibrations in the pulse. To identify the optimal operating point, a parametric analysis was performed by varying the IFBW in steps (100 kHz, 200 kHz, 300 kHz, 500 kHz, and 1 MHz), maintaining a constant acquisition time of 60 s. The results are summarized in Figure 23 and Table 3. Figure 23 (left) illustrates the evolution of the phase signal in the time domain. It can be seen that at high IFBW frequencies (e.g., 1 MHz—purple curve), the signal is affected by visible high-frequency noise, which overlaps the breath waveform. At the opposite end of the spectrum, for IFBW of 100 kHz (black curve), the signal is extremely clean, but its amplitude appears slightly attenuated.

The quantitative analysis of the phase shift, presented in Table 3, indicates a non-linear behavior. Maximum performance is not achieved at the ends of the range, but in a median area. According to the data, the IFBW = 300 kHz setting provides the best system sensitivity. At this point, the phase shift associated with breathing reaches a maximum of 90 degrees, and that associated with the pulse reaches 6 degrees. At IFBW < 300 kHz, despite the reduced noise, it appears to introduce slight smoothing of transient peaks, reducing the effective amplitude (69° at 100 kHz). At IFBW > 300 kHz, the phase noise increases significantly, degrading the SNR. At 1 MHz, the detected pulse amplitude drops dramatically to only 2 degrees, being drowned out by the system’s background noise.

In conclusion, 300 kHz represents the ideal compromise between sampling rate and noise suppression for this experimental setup, ensuring the highest acquisition stability and maximizing the detectability of heartbeats.

### 4.3. The Impact of Acquisition Time at a Fixed IFBW of 300 kHz on Detection Sensitivity

The last parameter investigated in the process of improving the detection system’s sensitivity is the acquisition window duration. Although a longer measurement duration theoretically provides higher spectral resolution, in the case of physiological monitoring, constraining factors such as involuntary movements of the subject and heart rate variability are involved, which can degrade signal integrity over long intervals. To determine the optimal duration, measurements were performed for four distinct time intervals: 30 s, 60 s, 120 s, and 240 s, keeping the previously set parameters constant (N_points = 100,000, IFBW = 300 kHz).

**Figure 24:**
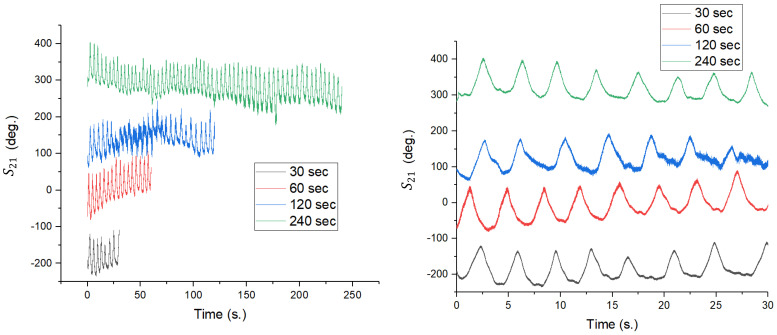
Evolution of the $S_{21}$ signal in the time domain for different acquisition durations. (**left**) The full graph shows a significant baseline drift for the 240 s acquisition (green); (**right**) Detail of the first 30 s, demonstrating the consistency of the waveform over short durations.

The time domain analysis, shown in Figure 24 (left), highlights the nature of the baseline drift. For short durations (30 s—black line), the signal may be more stable, and the respiratory oscillations may be more centered and uniform. As the duration increases to 120 s (blue) and especially to 240 s (green), signal drift trend/trends (a decreasing slope or irregularities) can be observed, most likely caused by the subject’s postural relaxation or involuntary micro-movements accumulated over time. This instability in the time domain complicates further processing and can introduce phase errors. The detailed image in Figure 24 (right) shows that although the waveform of the respiration is maintained, the signal quality (where the pulse is hidden) tends to be affected by the noise produced by movement during long measurements.

**Figure 25:**
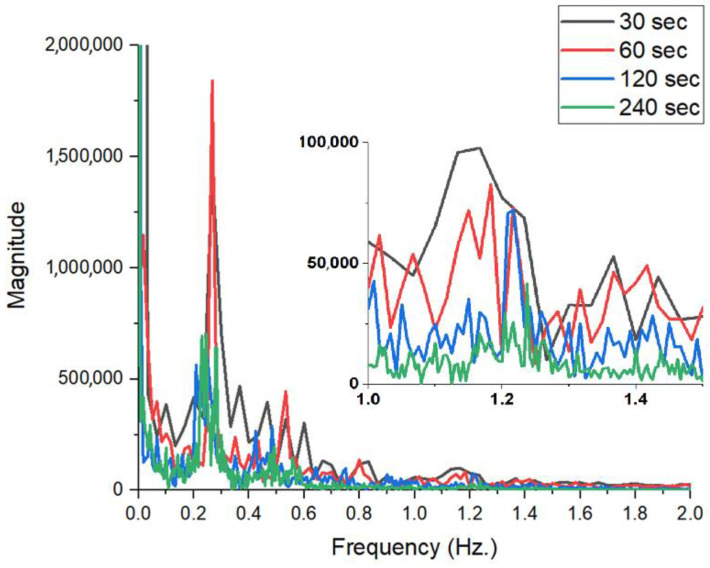
FFT amplitude spectrograms for the four acquisition lengths analyzed.

The impact on sensitivity is visible in the spectral analysis in Figure 25. Regarding breathing, all durations correctly detect the respiratory frequency. However, at 30 s (black line), the spectral resolution is weaker, with the main lobe being wider. The 60 s duration (red line) provides a much sharper and better-defined spectral peak.

Regarding pulse (inset image), this is where the critical difference appears. Contrary to theoretical expectations, very long durations (120 s and 240 s) show a decrease in pulse amplitude (the blue and green lines in the inset are well below the red and black lines). This phenomenon can be explained by the non-stationary nature of the human heart rate: over the course of 4 min, the heart rate varies slightly, which leads to the spectral energy being “spread” over a wider band, reducing the height of the detected peak.

Experimental analysis indicates that a 60 s acquisition is the optimal compromise for this system. This maximizes the SNR and pulse detection accuracy while avoiding the issues caused by subject motion and drift associated with long monitoring sessions.

## 5. Conclusions

This work presented the design, optimization, and comprehensive experimental validation of a high-gain 12 GHz 4 × 4 microstrip patch antenna array dedicated to non-contact vital signs monitoring based on phase variations in the $S_{21}$ transmission coefficient measured with a vector network analyzer. The antenna was progressively refined through coaxial feeding, slot-based impedance control, stepped transmission line matching, and mitered bends, resulting in a compact planar structure with a measured gain above 17 dBi, excellent impedance matching (−26 dB at 12 GHz), and a total efficiency close to 74%. Far-field measurements confirmed a very good agreement between simulated and experimental radiation characteristics, validating the electromagnetic design approach.

Beyond classical antenna characterization, a key contribution of this study lies in the systematic validation of the antenna array in a realistic $S_{21}$ phase-based vital signs monitoring scenario. The proposed array was experimentally compared with three fundamentally different antenna topologies: a commercial broadband biconical antenna, a single microstrip patch radiator, and a low-gain multi-lobular MIMO antenna. This multi-step validation strategy enabled a clear separation of the effects of antenna gain, beamwidth, and spatial selectivity on detection sensitivity. The results demonstrated that while respiration can be reliably detected even with low-gain or omnidirectional antennas, robust heartbeat detection—associated with micrometer-scale chest or arterial motion—requires high directivity and effective aperture. The 4 × 4 patch array consistently outperformed the biconical and single-patch antennas in terms of phase excursion and signal-to-noise ratio in the cardiac frequency band, while comparisons with the MIMO antenna highlighted the trade-off between spatial coverage and phase sensitivity.

Importantly, the experimental findings of this work confirm and extend the conclusions previously obtained by the authors in the article [34]. In that study, a computational parametric analysis demonstrated that antenna gain, directivity, impedance stability, and mutual coupling play a dominant role in determining vital-sign detection sensitivity, often exceeding the influence of signal processing techniques. The present work provides direct experimental validation of those conclusions at a higher operating frequency, using a different measurement paradigm based on $S_{21}$ phase tracking rather than classical Doppler demodulation. In particular, the necessity of high-gain, well-matched antennas for reliable heartbeat detection is fully confirmed, while the present results further show that receive-side directivity and phase stability are critical factors in $S_{21}$-based systems.

In addition to antenna design, this study demonstrated that the sensitivity of $S_{21}$ phase-based vital signs monitoring is jointly governed by the configuration of the VNA acquisition parameters, which must be optimized together with the antenna system. A high temporal resolution, obtained through a large number of acquisition points, was shown to be mandatory for accurately capturing transient cardiac micro-motions. The intermediate frequency bandwidth, IFBW, was identified as a critical parameter, with an optimal value around 300 kHz providing the best compromise between phase noise suppression and acquisition speed. Furthermore, the acquisition duration was shown to directly affect signal stationarity: while longer recordings increase nominal spectral resolution, excessive durations introduce baseline drift and heart rate variability effects that degrade pulse detectability.

Overall, the results demonstrate a strong consistency between computational predictions and experimental observations across different sensing architectures. The study confirms that antenna design is a primary enabling factor in non-contact vital signs monitoring and shows that the 12 GHz band represents a viable and effective operating point for high-sensitivity $S_{21}$ phase-based physiological sensing in controlled indoor environments. Future work will focus on extending the proposed approach to real-time implementations, multi-subject scenarios, and more complex propagation conditions, including through-clothing and through-obstacle measurements.

## Figures and Tables

**Table 1:** Evolution of 4 × 4 network performance depending on the type of applied optimization.

Configuration	Feed Type	Slots	Stepped Transmission Line Adaptation	Mitered Bends	Gain (dBi)	$S_{11}$ (dB)	Efficiency (%)	Observations
Initial (microstrip)	Microstrip	No	No	No	16.3	−24.73	71	Slightly deviated main lobe
With coaxial feed	Coaxial	No	No	No	16.5	−17.46	65.09	Improved symmetry
With slots	Coaxial	Yes	No	No	17.1	−20.48	70.82	Increased directivity
With stepped adaptation	Coaxial	Yes	Yes	No	17.8	−18.23	73.57	Reduced reflections, precise beam
Final	Coaxial	Yes	Yes	Yes	17.8	−26.06	73.96	Optimal performance achieved

**Table 2:** Comparative analysis of detected pulse magnitude as a function of the number of points and IFBW set on the VNA.

IFBW (kHz)	$N_{points}$	Pulse Magnitude
100	${20\times 10}^{3}$	${16\times 10}^{3}$
100	${100\times 10}^{3}$	${50\times 10}^{3}$
500	${20\times 10}^{3}$	${11\times 10}^{3}$
500	${100\times 10}^{3}$	${55\times 10}^{3}$
1000	${20\times 10}^{3}$	${20\times 10}^{3}$
1000	${100\times 10}^{3}$	${76\times 10}^{3}$

**Table 3:** Variation in phase shift measured for respiration *θ_resp_* and pulse *θ_pulse_* as a function of IFBW.

IFBW (kHz)	$\theta_{resp}$ (deg)	$\theta_{pulse}$ (deg)
100	69	4
200	84	5
300	90	6
500	57	4
1000	65	2

## Data Availability

The original contributions presented in this study are included in the article. Further inquiries can be directed to the corresponding author.
